# Supplementary material for: WNT5A is transported via lipoprotein particles in the cerebrospinal fluid to regulate hindbrain morphogenesis
Source: Nat Commun. 2019 Apr 2;10:1498. doi: 10.1038/s41467-019-09298-4 (PMC6445127; doi:10.1038/s41467-019-09298-4)
Supplement: Supplementary file 1 — Supplementary Information [file 41467_2019_9298_MOESM1_ESM.pdf]

Supplementary Information

WNT5A is transported via lipoprotein particles in the cerebrospinal fluid to regulate hindbrain morphogenesis.

Kaiser et al.

Supplementary Figure 1

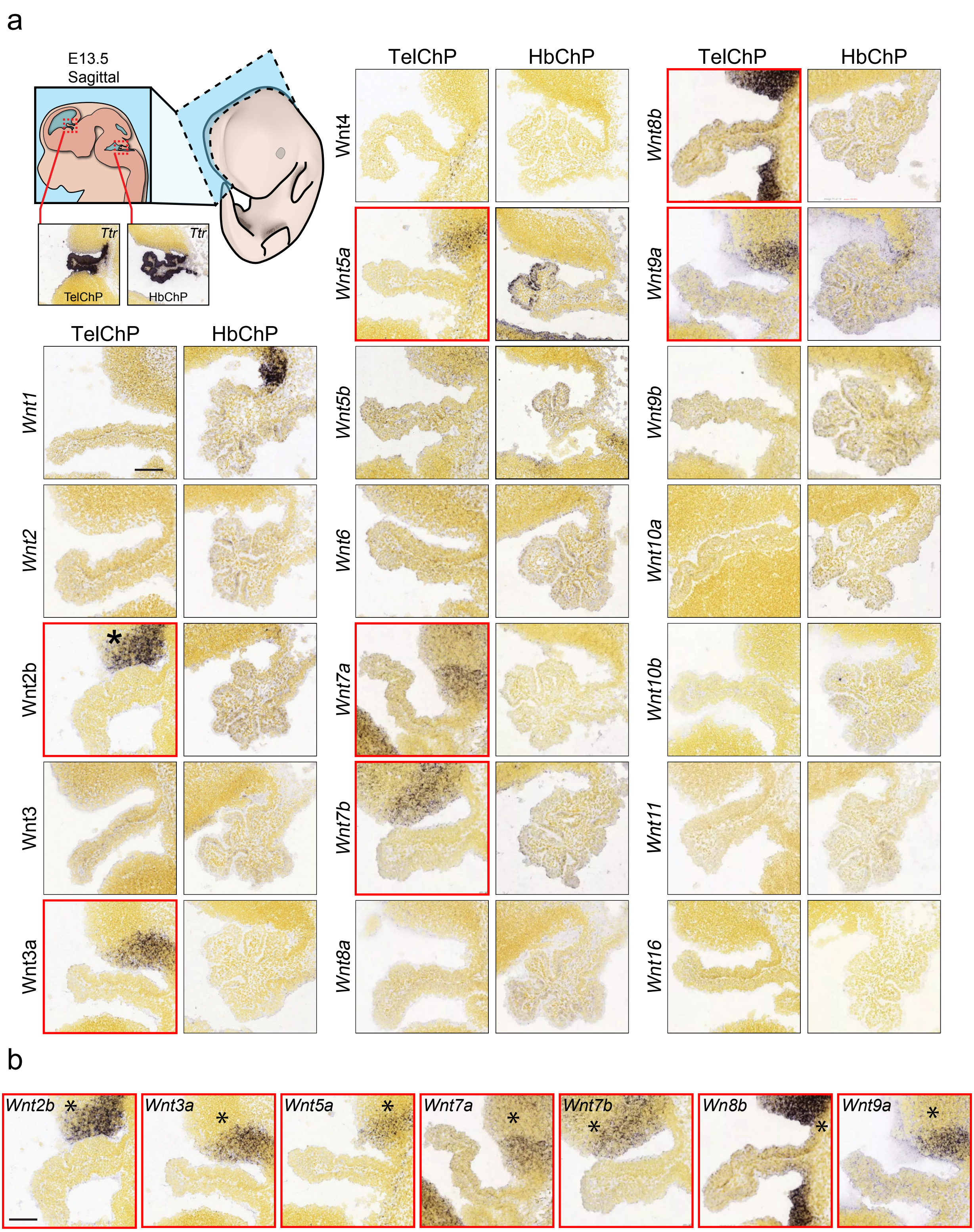

Supplementary Figure 1 Wnt genes expression in choroid plexus at E13.5
(a) Cartoon shows anatomical localization of ChPs depicted in the figure. Panel of the expression profiles for all Wnt genes at E13.5 in TelChP and HbChP. *Wnt5a* is the only gene with robust expression in HbChP epithelium, which is absent in TelChP epithelium. Scale bar represents 100 µm. (b) Among all analyzed Wnt genes, *Wnt2b*, *Wnt3a*, *Wnt5a*, *Wnt7a*, *Wnt7b*, *Wnt8b* and *Wnt9a* are expressed in Cortical Hem (CH; black asterisks, red frames) but not in either the TelChP or HbChP epithelium. Image credit: Allen Institute. Scale bar: 100 µm.

Supplementary Figure 2

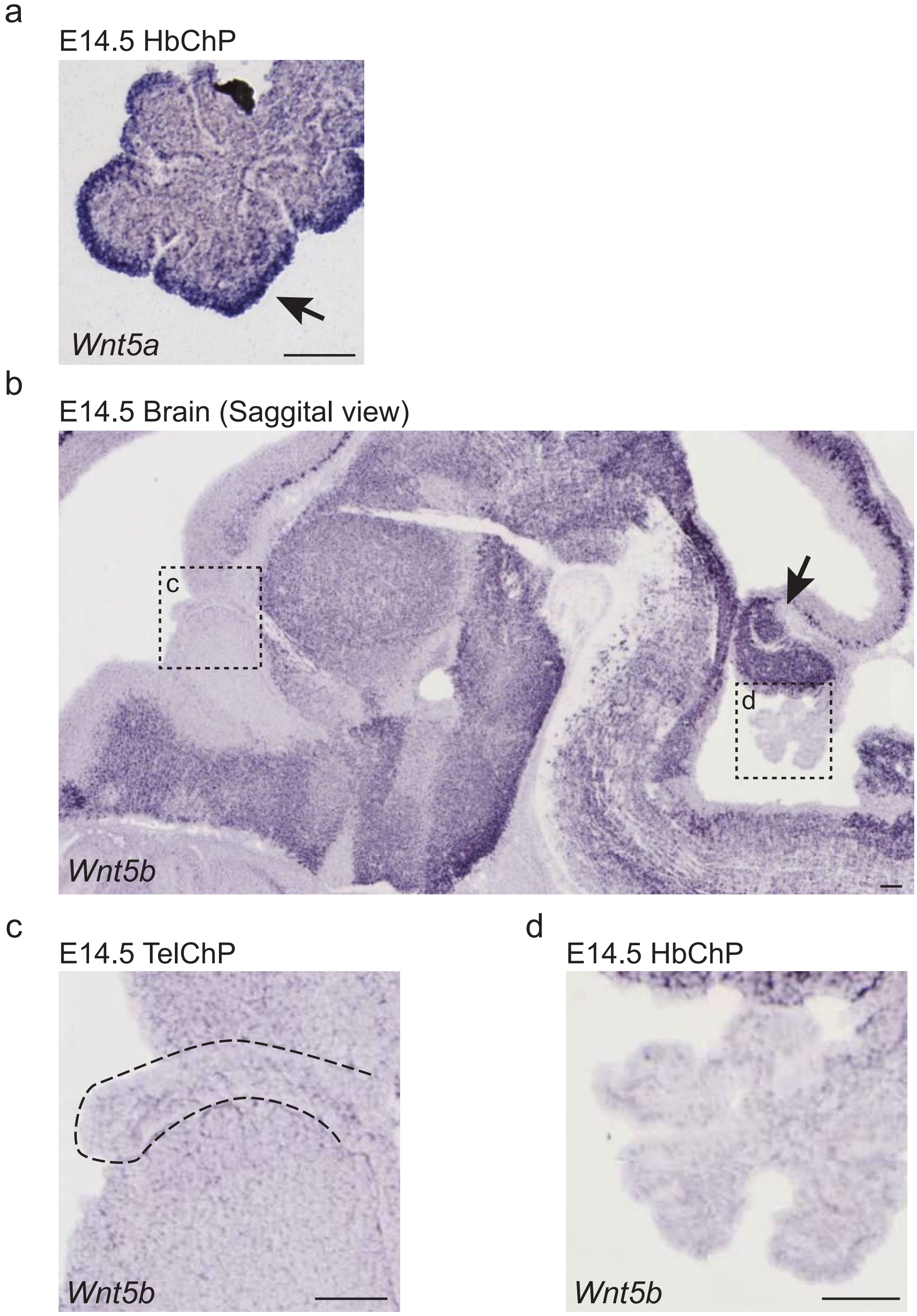

Supplementary Figure 2 Expression of Wnt5b in embryonic TelChP and HbChP

(a) Representative image of E14.5 sagittal section of mouse embryonic HbChP shows strong expression of *Wnt5a* in the HbChP epithelium (arrow). Expression level seems to be significantly higher compared to expression level of *Wnt5b* in HbChP. Image credit - http://www.eurexpress.org/ee. Scale bar: 100 µm.

(b-d) Representative image of E14.5 sagittal section of mouse embryonic brain (b) shows *Wnt5b* expression to be absent from both TelChP (c; dotted line) and HbChP (d). High expression of *Wnt5b* in developing cerebellum (arrow), region adjacent to HbChP, underlines absence or low level of the *Wnt5b* expression in the HbChP itself. Image credit - http://www.eurexpress.org/ee. Scale bar: 100 µm.

Supplementary Figure 3

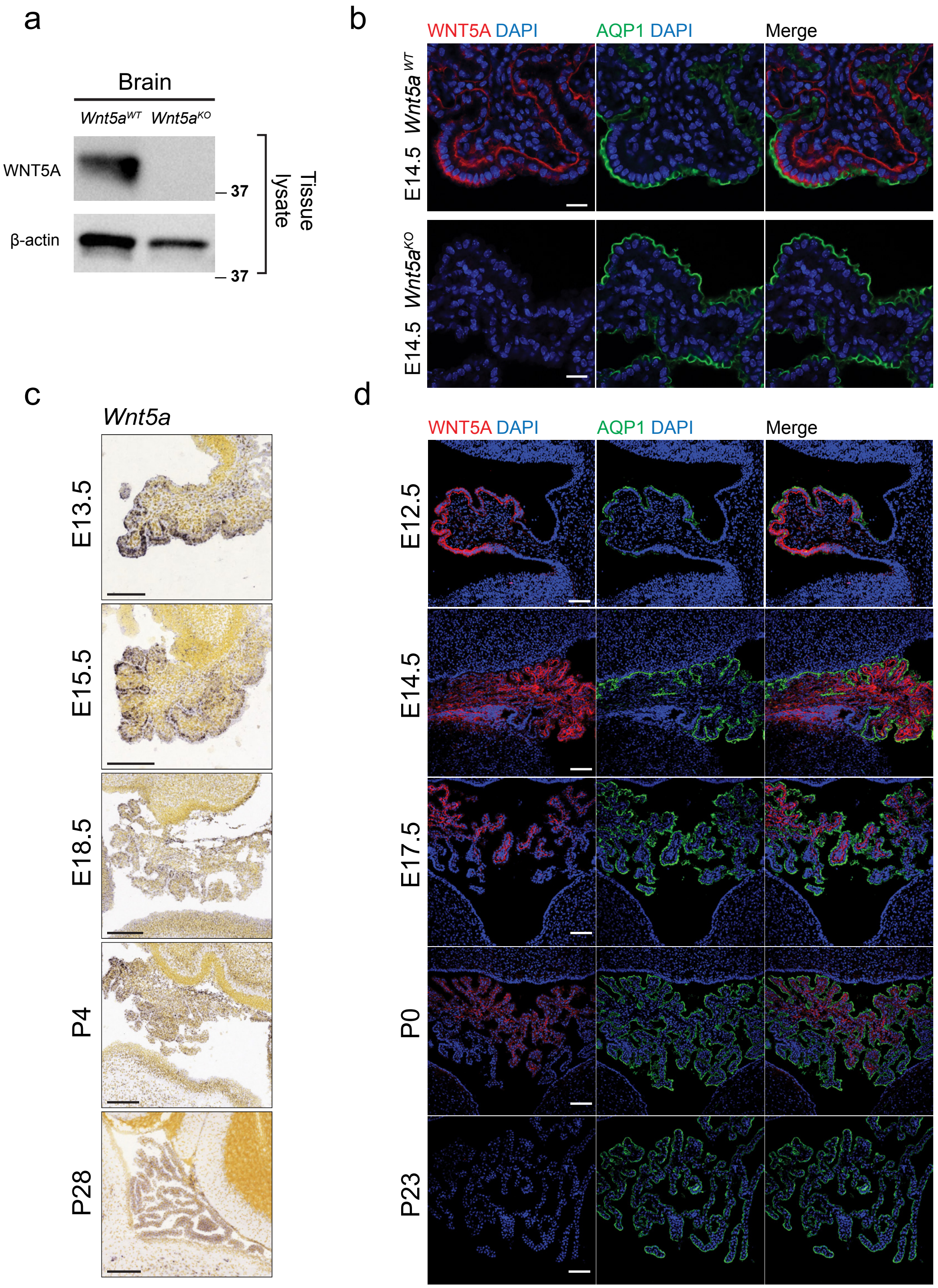

Supplementary Figure 3 Validation of WNT5A antibody and analysis of Wnt5a expression and production throughout mouse embryonic and postnatal development in HbChP

(a) Validation of WNT5A antibody for western blot analysis using brain tissue lysates from either Wnt5a$^{+/+}$ (*Wnt5a*^{WT}) and Wnt5a$^{-/-}$ (*Wnt5a*^{KO}) extracted at E16.5 , n=3. Signal for WNT5A is detected only in Wnt5a^{WT} confirming specificity of the antibody. β-actin serves as a loading control. (b) Validation of WNT5A antibody for immunofluorescence application, n=5. Signal for WNT5A can be detected only in *Wnt5a*^{WT} HbChP as compared to *Wnt5a*^{KO} HbChP. AQP1 is a marker of apical membrane of ChP epithelium. Scale bar: 20µm. (c) Panel indicating *Wnt5a* expression during embryogenesis, early (P4) and late postnatal stages (P28). Image credit: Allen Institute. Scale bar: 200µm and 100µm in case of E13.5 image. (d) Immunofluorescent analysis of WNT5A protein levels during embryogenesis, early (P0) and late postnatal stages (P23), n=3. WNT5A is completely lacking at P23 in accordance with the gene expression data. Scale bar: 100 µm.

Supplementary Figure 4

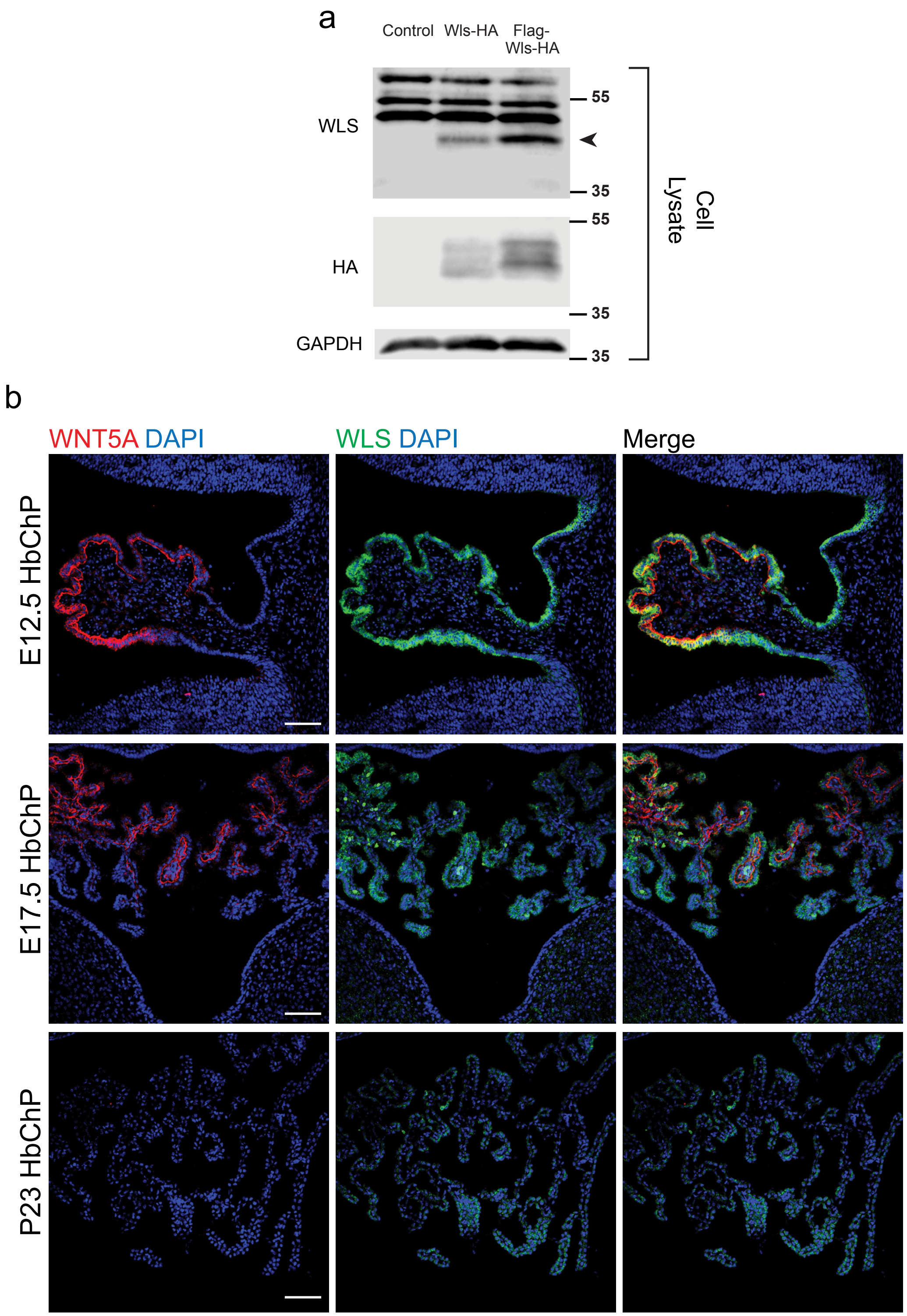

Supplementary Figure 4 Wls detection in HbChP during development and adulthood

(a) Western blot analysis of WLS antibody showing recognition of specific band corresponding to the size of band observed only in lysates of embryonic HbChP isolated at E14.5 and E17.5 (Figure 3c), n=3. Cell lysates used for analysis were obtained from HEK293T cells transfected with empty pcDNA3.1 vector (control), C-terminal HA-tagged *Wls* construct or C-Terminal HA-tagged construct with N-terminal FLAG tag. Cells were harvested 24h post transfection. β-actin serves as a loading control.

(b) WLS protein levels in HbChP analyzed at E12.5, E17.5 and P23 indicate reduction of WLS signal intensity over time, n=3. Scale bar: 100 μm.

Supplementary Figure 5

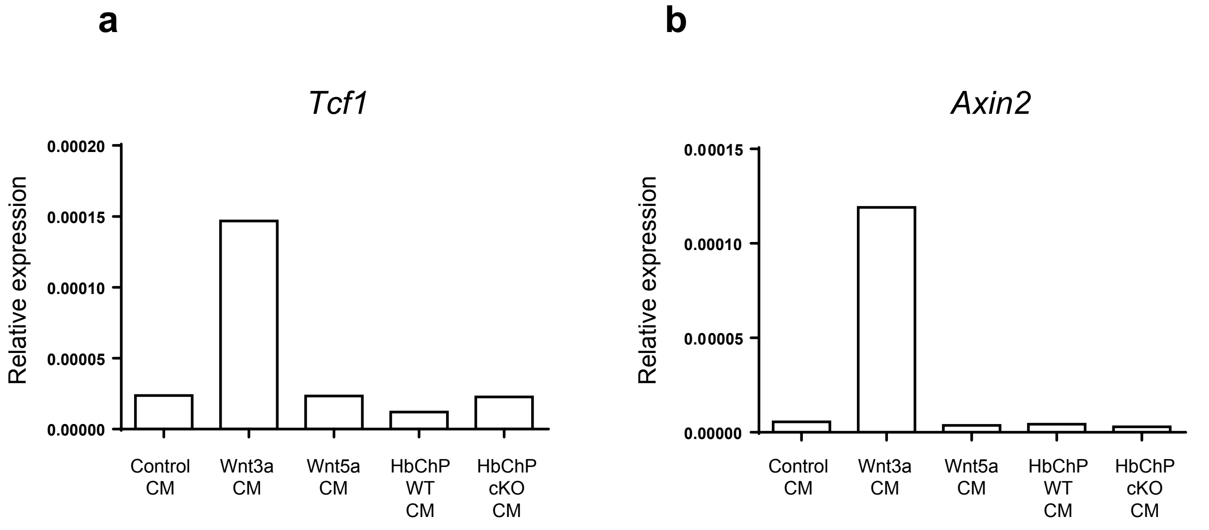

Supplementary Figure 5 Expression of canonical Wnt signaling target genes upon treatment with CM from HbChP primary culture

(a,b) Gene expression analysis by real-time qPCR of canonical Wnt signalling target genes (a) *Tcf1* and (b) *Axin2* upon treatment with various CM derived from either Wnt producing L-cells or $Wnt5a^{WT}$ and $Wnt5a^{cKO}$ ChP epithelium primary cultures (n=1). The expression levels for *Tcf1* and *Axin2* gene expression were normalized against expression level of *β-actin* in each condition. Source data are provided as a Source Data file.

Supplementary Figure 6

a

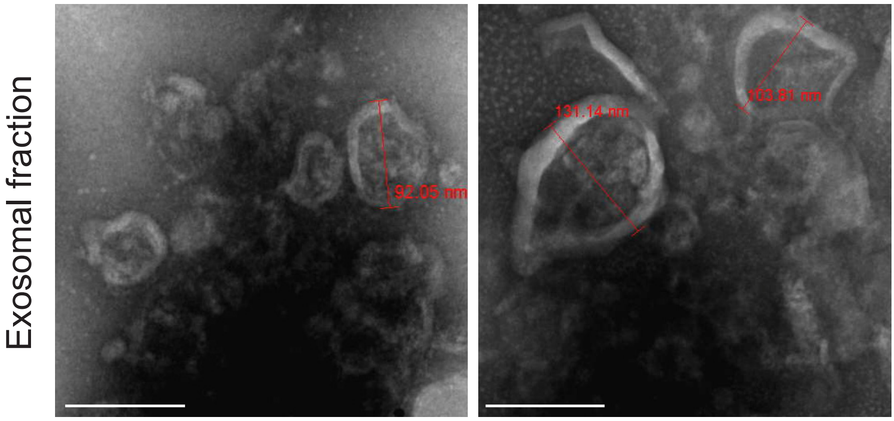

Supplementary Figure 6 Transmission electron microscopy analysis of exosomes
(a) Illustrative transmission electron microscopy (TEM) images of exosomes found in exosomal fraction obtained via ultracentrifugation isolation. TR-CSFB cells were cultivated for 48h at high confluency and collected CM has been subsequently subjected to ultracentrifugation to isolate purified fraction of exosomes, n=4. Depicted structures display characteristic features of exosomes, e.g. cup-shaped form, lipid bilayer and size similar to the expected size range. Scale bar: 100nm.

Supplementary Figure 7

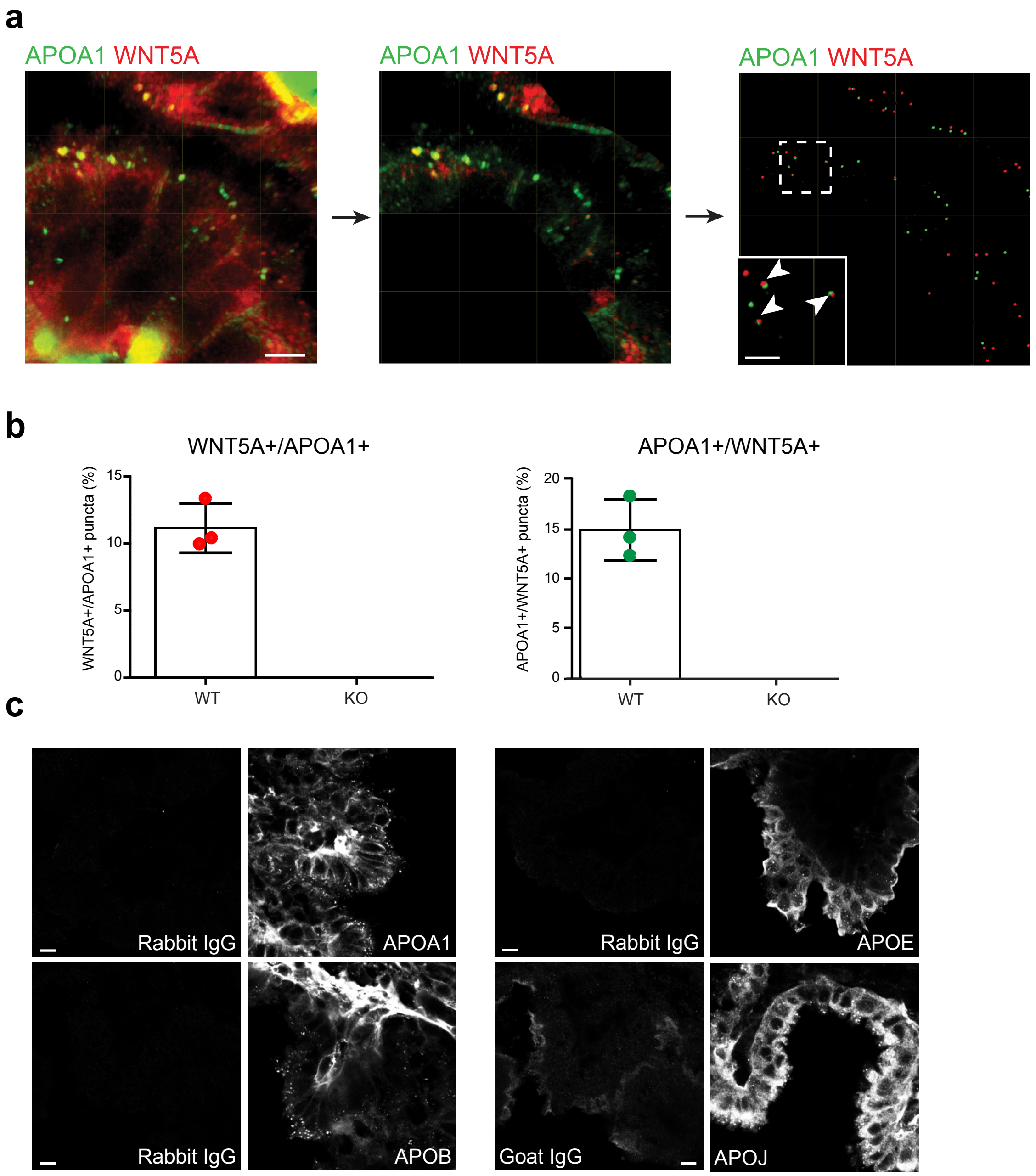

Supplementary Figure 7 Quantification analysis of WNT5A signal overlap

(a) Workflow of image processing for the signal quantification resulting in the generation of puncta that have been used in downstream analysis. Only WNT5A signal present in most apical region of analyzed HbChP epithelium has been used for the analysis. Arrowheads indicate dots with overlapping immunostaining signal. Scale bar: 5μm. Inset scale bar: 2μm.

(b) Validation of signal specificity by direct comparison of WNT5A+ dots stained for APOA1 (left) and APOA1+ dots stained for WNT5A (right) positive dots between *Wnt5a*^{WT} and *Wnt5a*^{KO} samples in HbChP epithelium at E14.5. Graph shows n=3 biologically independent samples; error bars represent mean ± s.d.

(c) Representative pictures for apolipoprotein antibodies staining with corresponding IgG serving as negative control to determine specificity of immuostaining signal for all the apolipoprotein antibodies used for immunostaining analysis (e.g. APOA1, APOB, APOE and APOJ) that have been used for quantitative analysis of WNT5A signal overlap, n=3. Scale bar: 10μm.

Source data are provided as a Source Data file.

Supplementary Figure 8

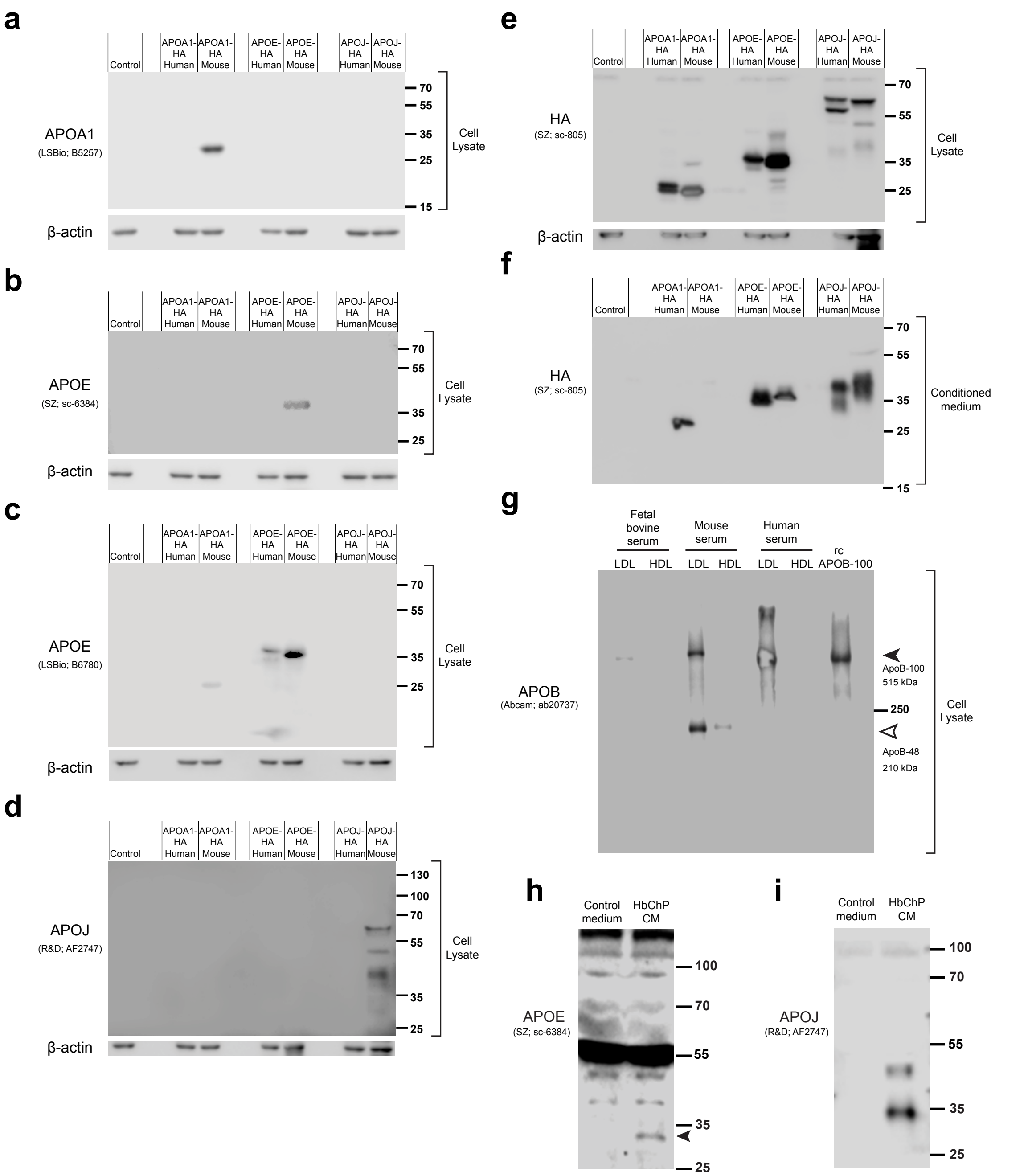

Supplementary Figure 8 WB validation of antibodies

(a-f) HEK293T cells were transfected either with empty pcDNA3.1 vector (control) or with various constructs coding for human and mouse apoliproteins,e.g. APOA1, APOE and APOJ, with HA C-terminal tag, n=3. Cells and CM were harvested 24h post transfection.

(a) Western blot analysis of APOA1 antibody (LSBio; B5257) showing its specificity only for mouse APOA1 protein from cell lysate. β-actin serves as a loading control.(b) Western blot analysis of APOE antibody (SZ; sc-6384) showing its specificity only for mouse APOE protein from cell lysate. β-actin serves as a loading control.(c) Western blot analysis of APOE antibody (LSBio; B6780) showing its specificity being mostly restricted to mouse APOE protein with low degree of cross-reactivity observed for human APOE and mouse APOA1 protein from cell lysate. β-actin serves as a loading control.

(d) Western blot analysis of APOJ antibody (R&D; AF2747) showing its specificity mostly for mouse APOJ protein from cell lysate (signal for human APOJ is much weaker as compared to mouse APOJ, data not shown). β-actin serves as a loading control. (e) Western blot analysis of the expression of all the tested HA-tagged apolipoprotein constructs. β-actin serves as a loading control.

(f) Western blot analysis of the CM obtained from transfected cells 24h post transfection confirming active secretion of all tested HA-tagged apolipoprotein constructs except for mouse APOA1. (g) Western blot analysis of APOB antibody (Abcam; ab20737) showing its specificity for human and mouse forms of APOB obtained from corresponding sera. Tested antibody also recognizes purified mouse APOB-100 recombinant protein (rcAPOB-100). 2 distinct bands observed at different size correspond to 2 subunits of APOB apolipoprotein; e.g. APOB-48 (210 kDa, empty arrowhead) and APOB-100 (515 kDa; arrowhead; UniProt accesion code: P04114; [https://www.uniprot.org/uniprot/P04114]). Validation of (h) APOE and (i) APOJ antibodies specificity for apolipoproteins secreted from HbChP epithelial primary cultures. There is no cross-reactivity with the apolipoproteins of bovine origin as shown by absence of signal in the control medium.

Supplementary Figure 9

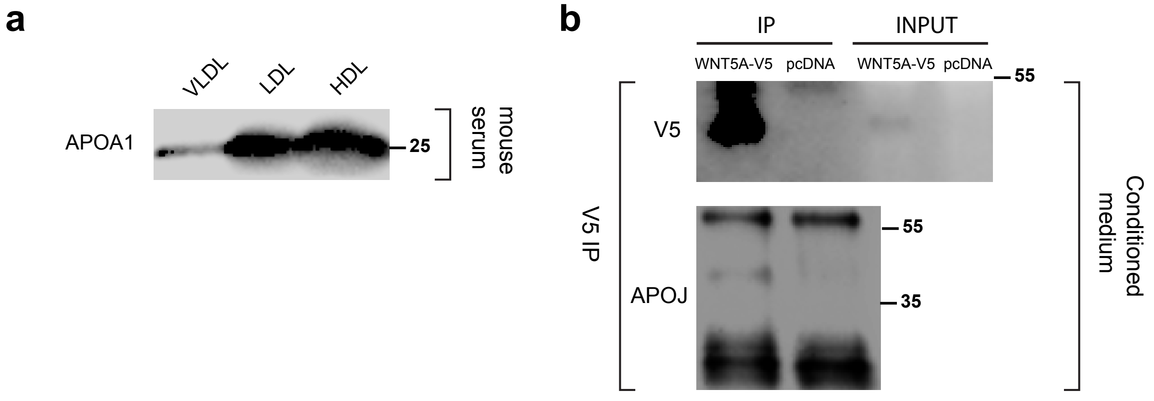

c

MS/MS analysis - Dataset of enriched proteins

Accesion	Protein name	Ratio of protein enrichment - sample vs control	Number of confirmed hits in HDL Proteome studies (*)	Number of confirmed hits in LDL Proteome studies (**)
A6QPP2	SERPIND1 protein	∞	6	0
Q9TT36	Thyroxine-binding globulin	∞	1	0
B2RYM3	Inter-alpha trypsin inhibitor, heavy chain 1	∞	3	0
F1N5M2	Vitamin D-binding protein	3,117 ↑	12	0
E1BMJ0	SERPING1	2,233 ↑	7	0
Q2KJF1	Alpha-1B-glycoprotein	1,747 ↑	9	1
F1MMK9	Protein AMBP	1,637 ↑	9	0
**P81644**	**Apolipoprotein A-II**	**1,620** ↑	**16**	**2**
Q05443	Lumican	1,562 ↑	3	0
P56652	Inter-alpha-trypsin inhibitor, heavy chain 3	1,461 ↑	2	0
**P15497**	**Apolipoprotein A-I**	**1,442** ↑	**17**	**4**

* HDL Proteome Watch (http://homepages.uc.edu/~davidswm/HDLproteome.html)

** LDL Proteome Watch (http://homepages.uc.edu/~davidswm/LDLproteome.html)

Supplementary Figure 9 Co-immunoprecipitation of secreted form of WNT5A-V5 and mass spectrometry analysis of interacting partners
(a) Mouse serum was subjected to discontinuous KBr gradient centrifugation and different isolated fractions (VLDL, LDL, and HDL) were analyzed for the presence of APOA1 as marker of HDL lipoprotein fraction, n=3. (b) Pull-down of WNT5A-V5 from CM cultured for 48h with either WNT5A-V5 or pcDNA transfected TR-CSFB cells. APOJ co-immunoprecipitated with WNT5A-V5. pcDNA serves as a negative control, n=3. (c) Mass spectrometry analysis of immunoprecipitation samples (IP WNT5A-V5) shown in (b) shows enrichment of proteins that have been confirmed by more than 3 independent proteomic studies to be associated with HDL proteome with exception of Thyroxine-binding globulin protein that has been detected only in a single HDL proteome analysis listed in the database HDL proteome Watch (http://homepages.uc.edu/~davidswm/HDLproteome.html).

Supplementary Figure 10

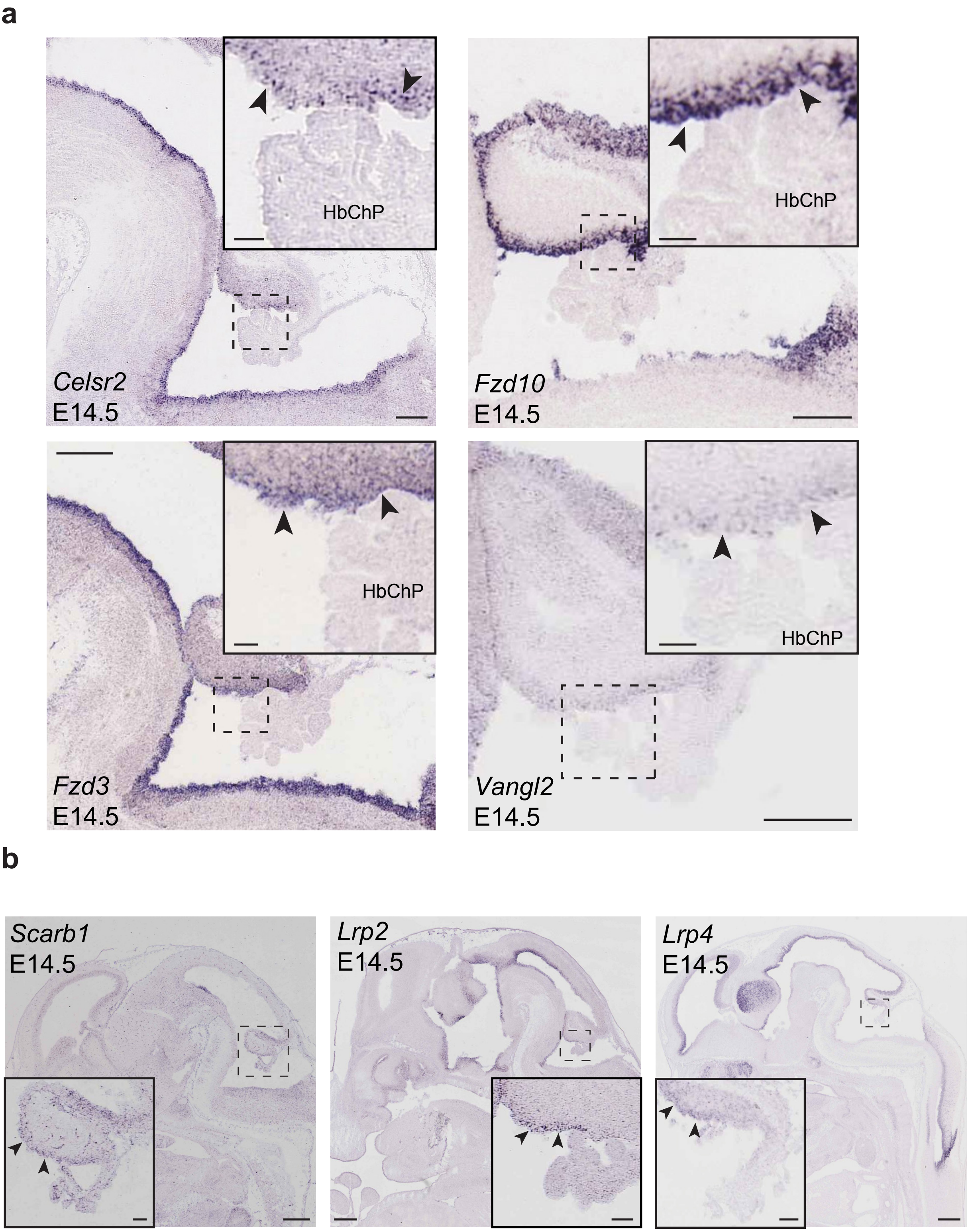

Supplementary Figure 10 Expression of Wnt signaling pathway components in ventricular zone of cerebellum
(a) Panel showing *in situ* analysis of gene expression for various Wnt pathway-related genes such as *Celsr2*, *Vangl2*, *Fzd3*, and *Fzd10*. Insets showing higher expression levels of highlighted genes in the ventricular zone of developing cerebellum (arrowheads). Data are adopted from http://www.eurexpress.org/ee/. Scale bar: 200 µm. Inset scale bar: 50 µm.
(b) Panel showing *in situ* analysis of gene expression for several genes encoding lipoprotein-receptors such as *Scarb1* (receptor for high-density lipoproteins), *Lrp2* and *Lrp4* (receptors involved in inhibition of Wnt signaling). Inset pictures showing increased expression of *Scarb1*, *Lrp2* and *Lrp4* in the ventricular zone of developing cerebellum (arrowheads). Data are adopted from http://www.eurexpress.org/ee/. Scale bar: 500 µm. Inset scale bar: 100 µm.

Supplementary Figure 11

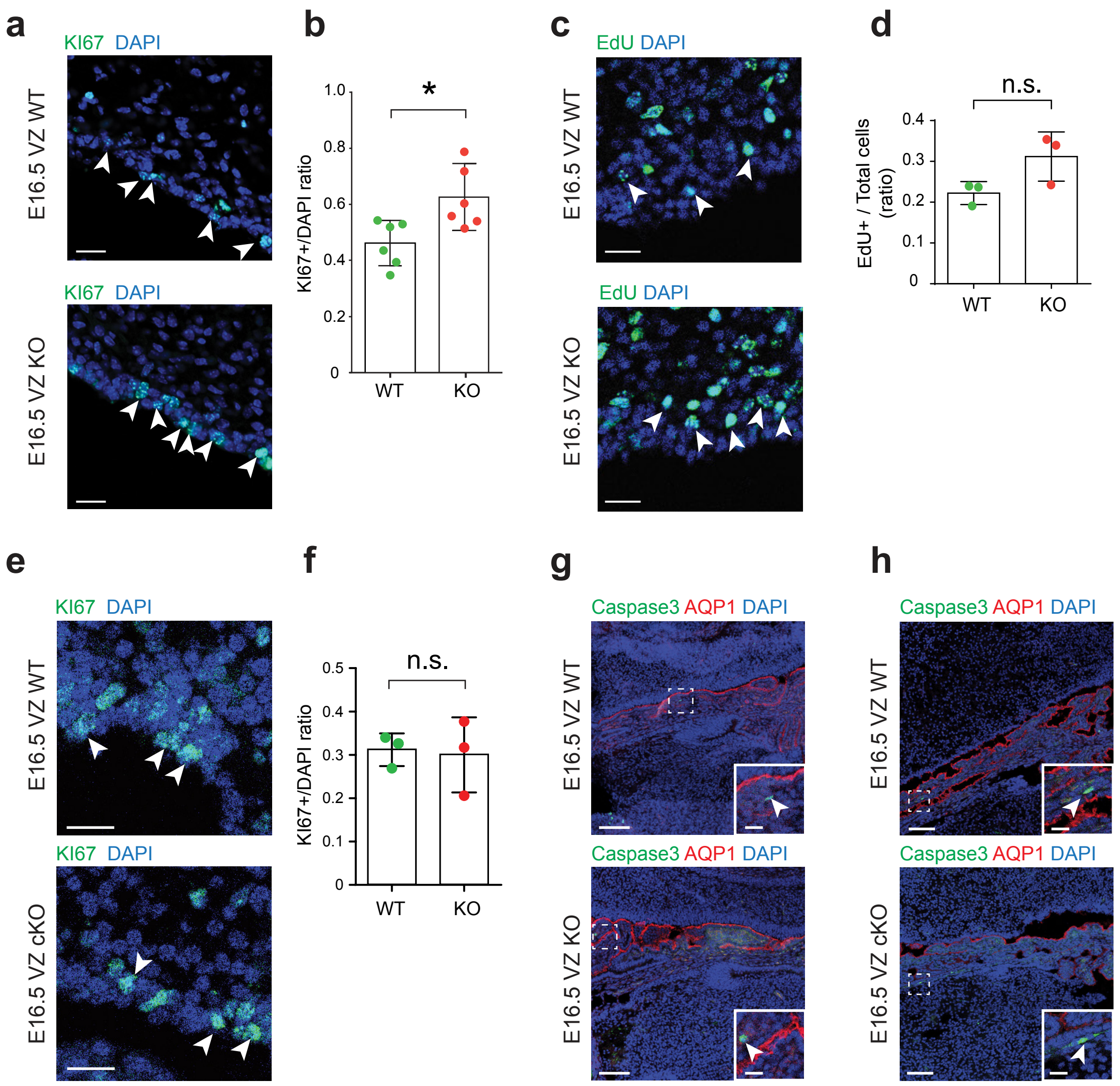

Supplementary Figure 11 Level of proliferation and apoptosis in developing cerebellum upon Wnt5a ablation

(a) Higher numbers of KI67+ cells in E16.5 *Wnt5a*^{KO} embryos (arrowheads) are detected in the most apical region of cerebral ventricular zone compared to WT embryos, n=6. Scale bar: 10 µm.

(b) Quantification of KI67+ corroborates these observations. Graph shows n=6 biologically independent animals; error bars represent mean ± s.d.; P values (two-tailed Student's t-test with unequal variance), * $P < 0.05$: E16.5 *Wnt5a*^{WT} vs *Wnt5a*^{KO} embryos: P = 0.0191.

(c) Representative image indicating modest increase in number of EdU+ cells in E16.5 *Wnt5a*^{KO} embryos (arrowheads) that can be detected in the most apical region of cerebral ventricular zone compared to WT embryos, n=3. EdU+ has been injected at E13.5 and embryos were harvested at E16.5 (72h post injection). Scale bar: 10 µm.

(d) Quantification of EdU+ cells corroborates observation showing minor increase in the number of EdU+ cells in the developing cerebellum. Graph shows n=3 biologically independent animals; error bars represent mean ± s.d. (two-tailed Student's t-test with unequal variance).

(e) Representative image of KI67+ cells in E16.5 *Wnt5a*^{WT} and *Wnt5a*^{cKO} embryos (arrowheads) detected in the most apical region of cerebral ventricular zone, n=3. Scale bar: 10 µm.

(f) Quantification of KI67+ cells highlights no change in the number of KI67+ cells between E16.5 *Wnt5a*^{WT} and *Wnt5a*^{cKO} embryos. Graph shows n=3 biologically independent animals; error bars represent mean ± s.d. (two-tailed Student's t-test with unequal variance).

(g,h) Representative images of Caspase3 staining in either E16.5 (g) *Wnt5a*^{KO} or (h) *Wnt5a*^{cKO} (h) show no difference in level of apoptosis in developing cerebellum as compared with *Wnt5a*^{WT} littermates, n=3. Inset images show presence of few apoptotic cells in the stromal region of adjacent HbChP (arrowheads). Scale bar: 100 µm. Inset scale bar: 20 µm.

Biological replicates are indicated in the graphs. Source data are provided as a Source Data file.

Supplementary Figure 12

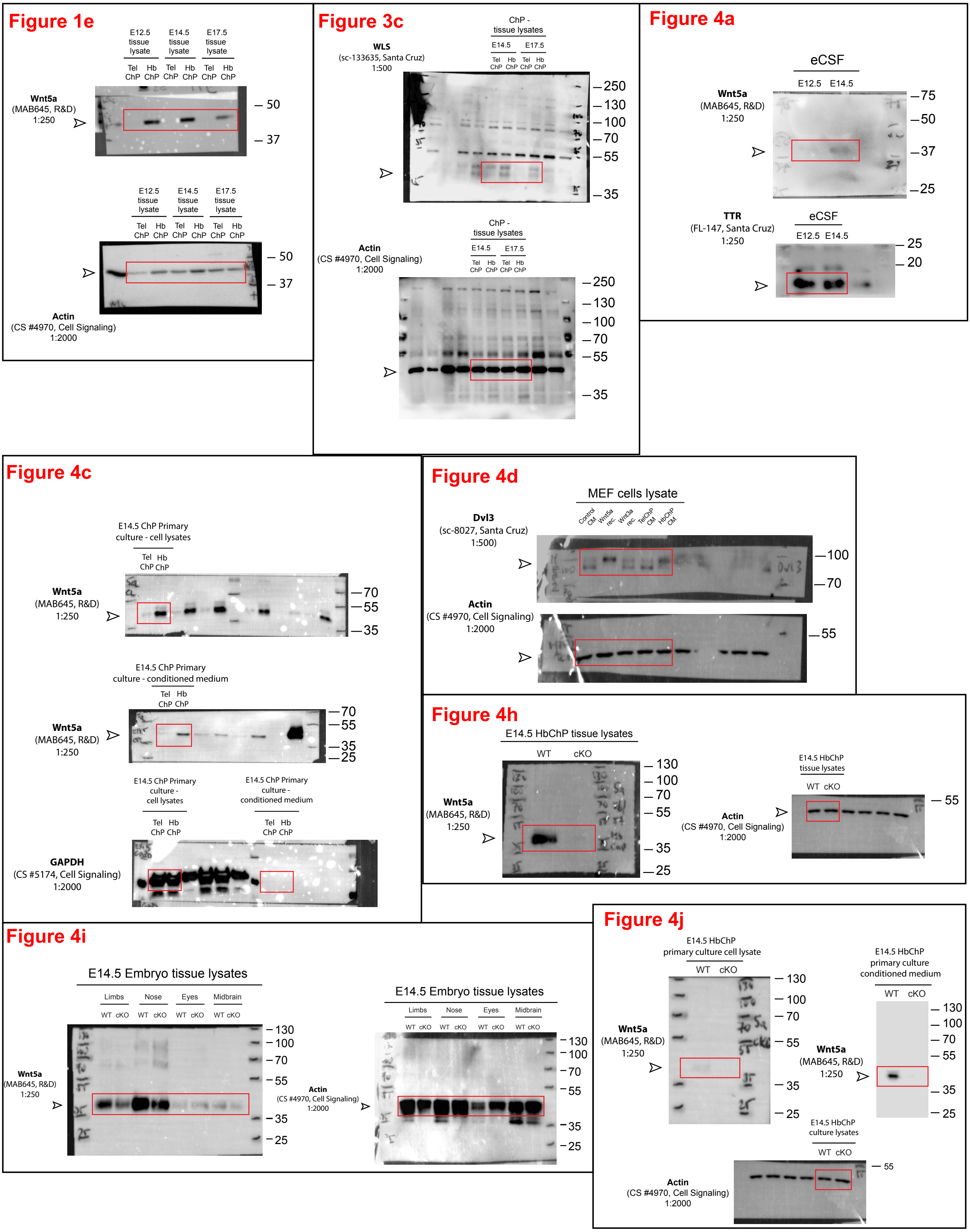

Supplementary Figure 12 Uncropped western blot images.
Cropped regions indicated in the main and supplementary figures are highlighted with red box.

Supplementary Figure 12

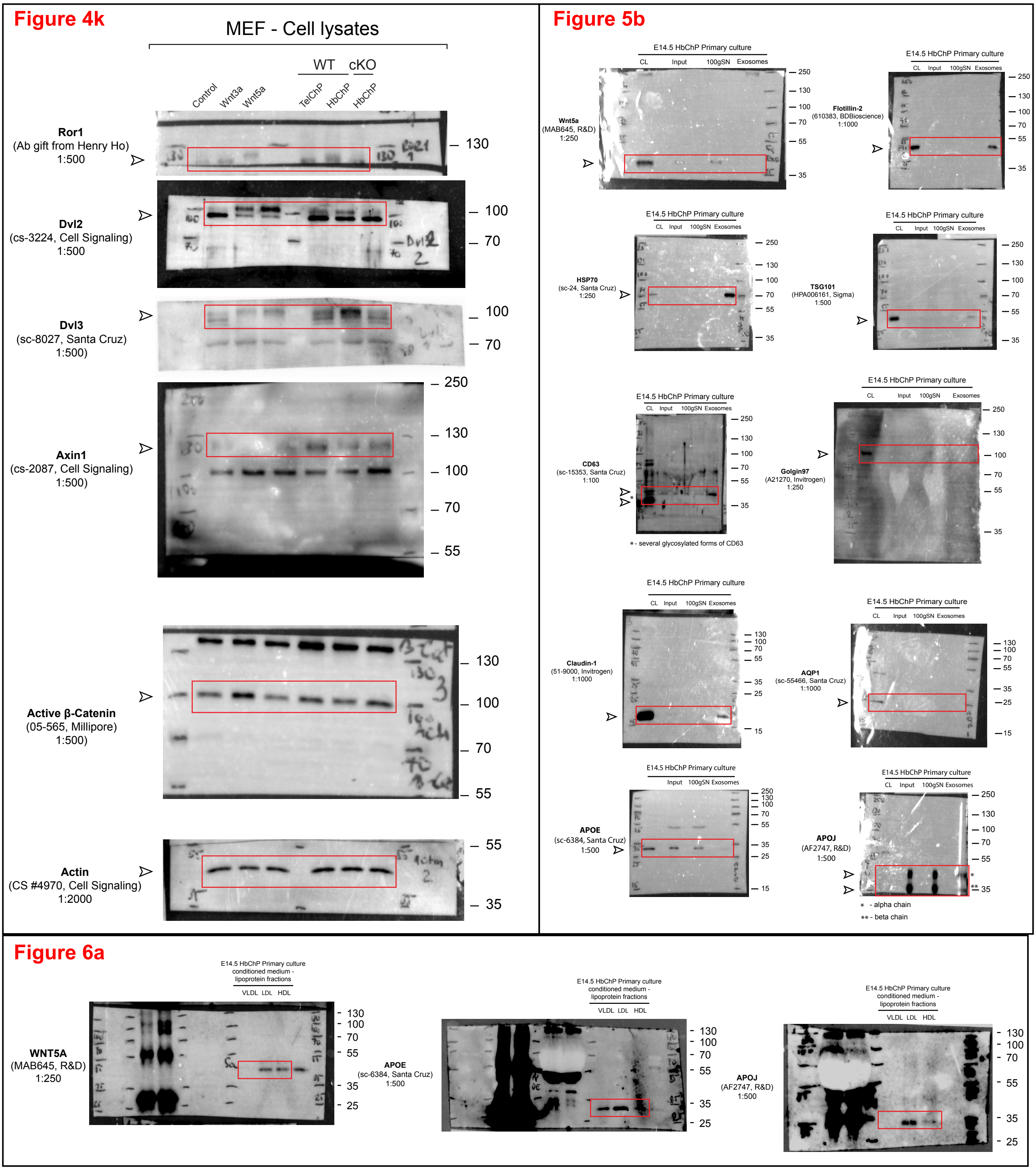

Supplementary Figure 12 Uncropped western blot images.
Cropped regions indicated in the main and supplementary figures are highlighted with red box.

Supplementary Figure 12

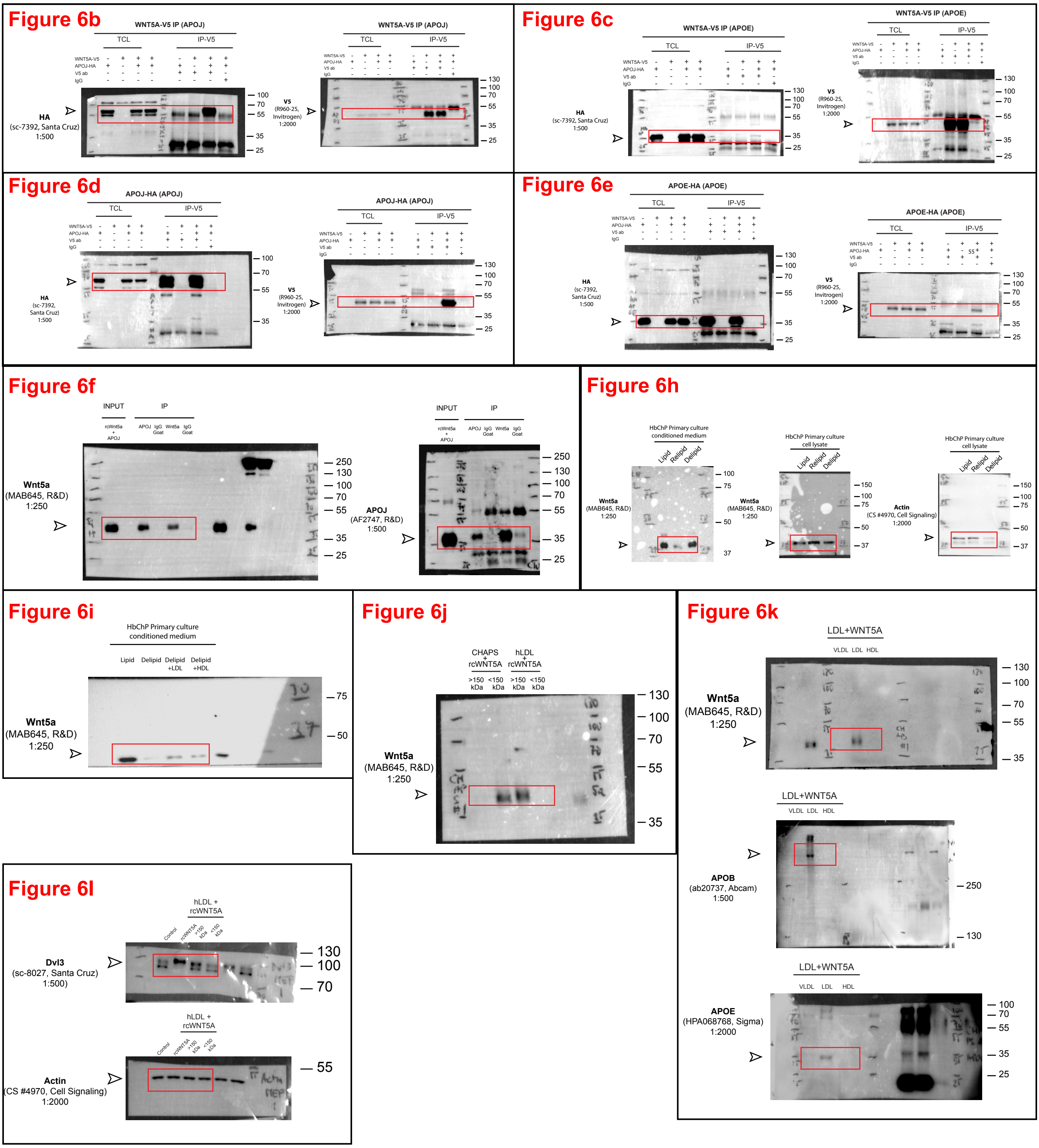

Supplementary Figure 12 Uncropped western blot images.
Cropped regions indicated in the main and supplementary figures are highlighted with red box.

Supplementary Figure 12

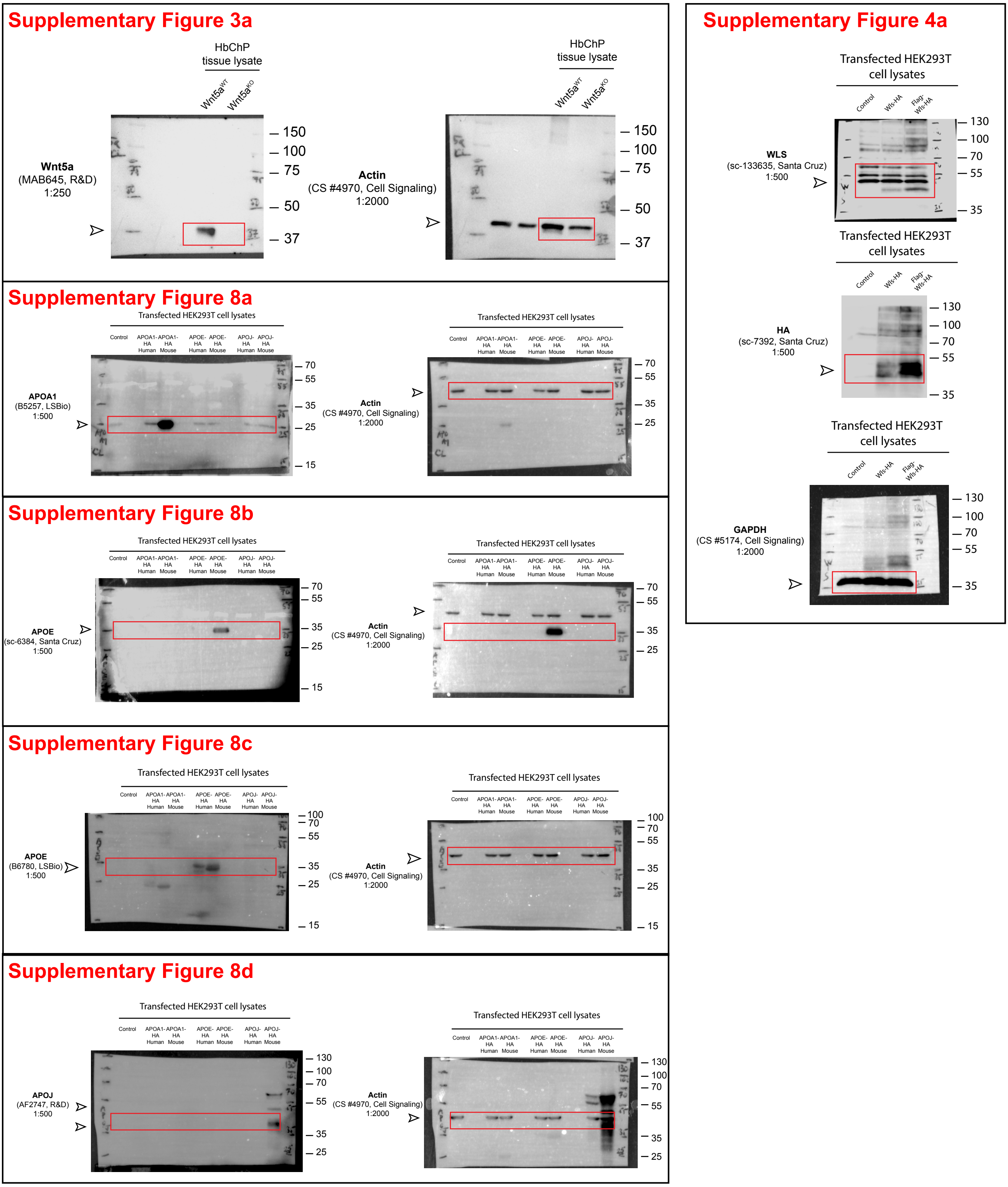

Supplementary Figure 12 Uncropped western blot images.
Cropped regions indicated in the main and supplementary figures are highlighted with red box.

Supplementary Figure 12

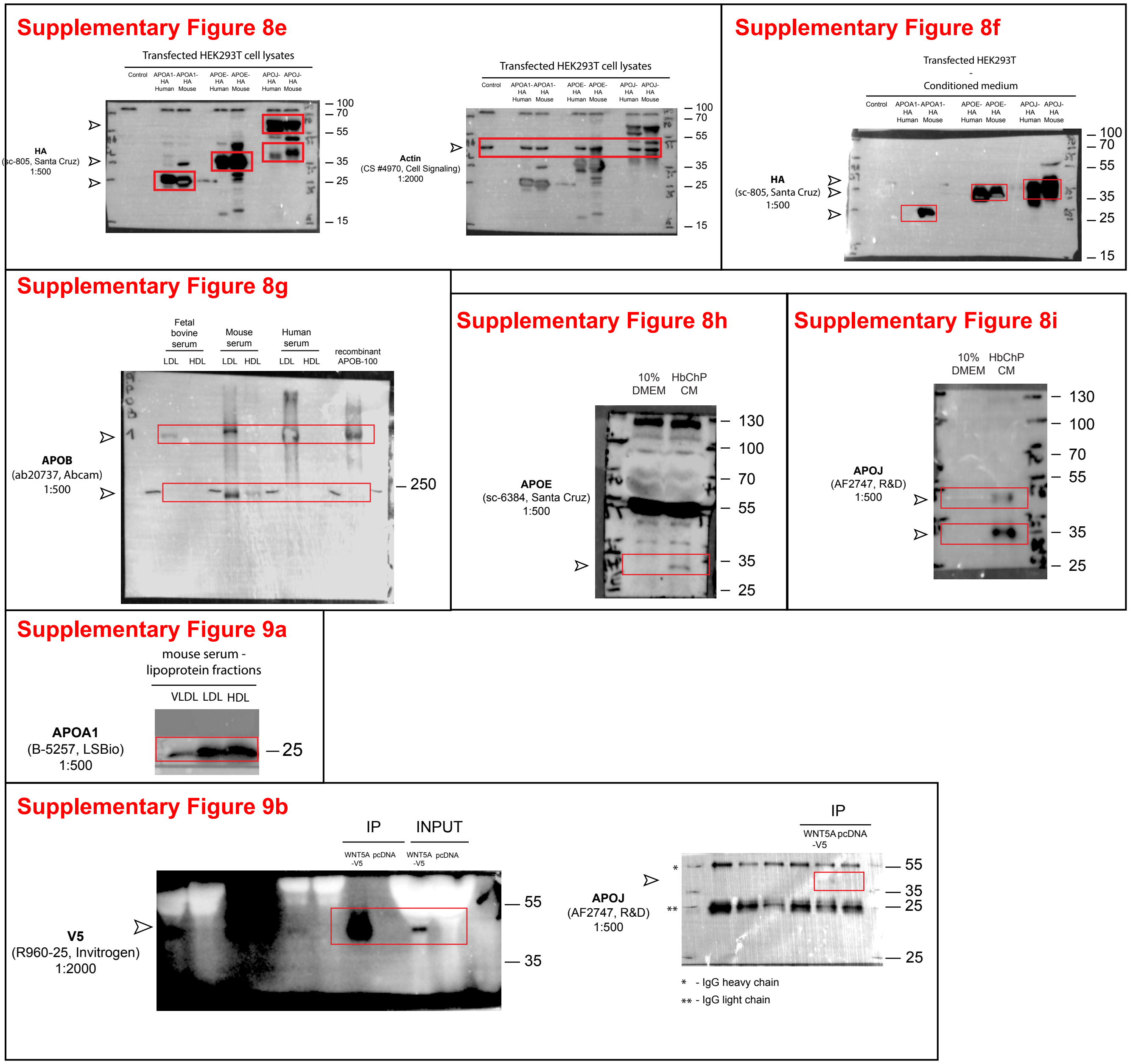

Supplementary Figure 12 Uncropped western blot images.
Cropped regions indicated in the main and supplementary figures are highlighted with red box.

Supplementary table 1

List of used reagents

Reagent	Manufacturer	Application
Recombinant Wnt3a	5036-WN-CF, R&D	Cell culture
Recombinant Wnt5a	645-WN-010, R&D	Cell culture, in vitro binding, IP
LGK 974 Porcupine inhibitor	974-02, StemRD	Cell culture
Laminin	L2020, Sigma	Cell culture
Mouse native Epidermal Growth Factor (EGF)	53003019, Thermo Fisher	Cell culture
Tamoxifen	T5648, Sigma	Animal work
Tissue-tek freezing medium	25608-930, VWR	Cryosectioning
Superfrost slides	10143352, Thermo Fisher	Cryosectioning
Click-iT™ EdU Alexa Fluor™ 555 Imaging Kit	C10338, Invitrogen	Immunofluorescence
EdU	E10187, Thermo Fisher	Immunofluorescence
Antigen retrieval solution	S1699, DAKO	Immunofluorescence
Mounting medium	S302380, DAKO	Immunofluorescence
Dynabeads	1000 4D, Invitrogen	IP
Protein G Sepharose 4 Fast Flow	17-0618-01, GE Healthcare	IP
Protease inhbitor cocktail	11836145001, Roche	IP
Dithiothreitol (DTT)	43819, Sigma	IP
Plasma native APOE (human)	SRP6303, Sigma	IP, in vitro binding
Plasma native APOJ (human)	RD162034025, Biovendor	IP, in vitro binding
Lipid Removal Agent (LRA)	13358-U, Sigma	Lipoprotein separation
Lightcycler 480 SYBR Green 1 Master Mix	04707516001, Roche	PCR
Pronase	10165921001, Sigma	Primary culture
Cytosine β-D-arabinofuranoside (AraC)	C1768, Sigma	Primary culture
3 kDa - Protein concentrator	PI88514S, Thermofisher	Protein separation
150 kDa - Protein concentrator	PI89922, Thermofisher	Protein separation
CHAPS	C3023, Sigma	Protein separation
DNase	M6101, Promega	RNA isolation
Fluorescent Multiplex Reagent Kit	320850, ACDbio	RNAscope
mWls probe	405011, ACDbio	RNAscope
mWnt5a probe	316791-C2, ACDbio	RNAscope
Recombinant APOB (mouse)	RPC003Mu02, Cloude-clone	WB
Mouse serum	10410, Thermofisher	WB
Immobilon-P membrane	IPVH00010, Merck	WB
Immobilon Western Chemiluminescent HRP Substrate	WBKLS0500, Merck	WB
Supersignal West Femto maximum sensitivity substrate	34035, Thermo Scientific	WB
human LDL	LP2-2MG, Millipore	Western, In vitro binding

Supplementary table 2

List of used constructs

Construct name	Source
pcDNA3.2	Wnt Open Source Kit (Kit#1000000022. Addgene)
WNT5A-V5 pcDNA3.2	Wnt Open Source Kit (Kit#1000000022, Addgene)
Human APOA-1-HA	HG10686-CY (SinoBiological)
Mouse APOA1-HA	MG53232-CY (SinoBiological)
Human APOE-HA	HG10817-CY (SinoBiological)
Mouse APOE-HA	MG51201-CY (SinoBiological)
Human APOJ-HA	HG11297-CY (SinoBiological)
Mouse APOJ-HA	MG50485-CY (SinoBiological)
hWls-HA	Kind gift from Mark von Zastrow
hFlag-Wls-HA	Kind gift from Mark von Zastrow

Supplementary table 3
List of used antibodies

Antibody	Manufacturer	Tested species	Application
β-Actin	CS-4970, Cell Signalling	Mouse	WB
APOA1	B-5257, LSBio	Mouse, Human	IF,WB
APOB	20737, Abcam	Mouse, Human	IF,WB
APOE	B6780, LSBio	Mouse, Human	IF
APOE	HPA068768, Sigma	Human	WB
APOE	SC-6384, Santa Cruz	Mouse, Human	WB
APOJ (Clusterin)	AF2747, R&D	Mouse, Human	IF, IP, WB
AQP1	SC-55466, Santa Cruz	Mouse, Human	IF, ICC,WB
AXIN1	CS-2087, Cell Signalling	Mouse	WB
Active β-Catenin	05-565, Millipore	Mouse	WB
Caspase 3	CS-9664, Cell Signalling	Mouse	IF
CD63	SC-15353, Santa Cruz	Mouse	IF,WB
Claudin-1	51-9000, Invitrogen	Mouse	WB
DVL2	CS-3216, Cell Signalling	Mouse	WB
DVL3	SC-8027, Santa Cruz	Mouse	WB
Flotillin-2	610383, BDBioscience	Mouse	WB
GAPDH	CS-5174, Cell Signalling	Mouse	WB
Golgin-97	A21270, Invitrogen	Mouse	WB
HA	ab9110, Abcam	tag	IP
HA	SC-7392, Santa Cruz	tag	WB
HSP70	SC-24, Santa Cruz	Mouse	WB
ROR1	kind gift from Henry Ho	Mouse	WB
TSG101	HPA006161, Sigma	Mouse	IF, WB
V5	R960-25, Invitrogen	tag	IP, WB
WLS	SC-133635, Santa Cruz	Mouse	IF,WB
WNT5A	AF645, R&D	Mouse, Human	IP
WNT5A	MAB645, R&D	Mouse, Human	IF, WB
ZO1	R26.4C, DSHB	Mouse	IF

Supplementary table 4

List of used primers

Primer	Sequence
Actin forward	AGCCATGTACGTAGCCATCC
Actin reverse	CTCTCAGCTGTGGTGGTGAA
Axin2 forward	GAAGAAATTCCATACAGGAGGAT
Axin2 reverse	GTCACTCGCCTTCTTGAAATAA
Tcf1 forward	AGCTTTCTCCACTCTACGAACA
Tcf1 reverse	AATCCAGAGAGATCGGGGGTC
Wls forward	TGTTGGAGGGATTCTTCTGG
Wls reverse	TTTGCTTCAATTTCCCTTGG
Wnt5a forward	AGGAGTTCGTGGACGCTAGA
Wnt5a reverse	ACTTCTCCTTGAGGGCATCG
