# Supplementary material for: WNT5A is transported via lipoprotein particles in the cerebrospinal fluid to regulate hindbrain morphogenesis
Source: Nat Commun. 2019 Apr 2;10:1498. doi: 10.1038/s41467-019-09298-4 (PMC6445127; doi:10.1038/s41467-019-09298-4)
Supplement: Supplementary file 3 — Reporting Summary [file 41467_2019_9298_MOESM3_ESM.pdf]

nature research

Corresponding author(s): Carlos Villlaescusa
Last updated by author(s): 2019-1-14

Reporting Summary

Nature Research wishes to improve the reproducibility of the work that we publish. This form provides structure for consistency and transparency in reporting. For further information on Nature Research policies, see Authors & Referees and the Editorial Policy Checklist.

Statistics

For all statistical analyses, confirm that the following items are present in the figure legend, table legend, main text, or Methods section.

n/a	Confirmed	
☐	☒	The exact sample size (n) for each experimental group/condition, given as a discrete number and unit of measurement
☐	☒	A statement on whether measurements were taken from distinct samples or whether the same sample was measured repeatedly
☐	☒	The statistical test(s) used AND whether they are one- or two-sided<br>*Only common tests should be described solely by name; describe more complex techniques in the Methods section.*
☐	☒	A description of all covariates tested
☐	☒	A description of any assumptions or corrections, such as tests of normality and adjustment for multiple comparisons
☐	☒	A full description of the statistical parameters including central tendency (e.g. means) or other basic estimates (e.g. regression coefficient) AND variation (e.g. standard deviation) or associated estimates of uncertainty (e.g. confidence intervals)
☐	☒	For null hypothesis testing, the test statistic (e.g. F, t, r) with confidence intervals, effect sizes, degrees of freedom and P value noted<br>*Give P values as exact values whenever suitable.*
☒	☐	For Bayesian analysis, information on the choice of priors and Markov chain Monte Carlo settings
☒	☐	For hierarchical and complex designs, identification of the appropriate level for tests and full reporting of outcomes
☒	☐	Estimates of effect sizes (e.g. Cohen's d, Pearson's r), indicating how they were calculated

Our web collection on statistics for biologists contains articles on many of the points above.

Software and code

Policy information about availability of computer code

Data collection	FluoView FV1000, Zeiss ZEN 2.0, LightCycler® 480 Software, Version 1.5, Vilber FUSION software (western blot)
Data analysis	Graphpad Prism 6.0, Bitplane Imaris 9.0, ImageJ 1.52

For manuscripts utilizing custom algorithms or software that are central to the research but not yet described in published literature, software must be made available to editors/reviewers. We strongly encourage code deposition in a community repository (e.g. GitHub). See the Nature Research guidelines for submitting code & software for further information.

Data

Policy information about availability of data

All manuscripts must include a data availability statement. This statement should provide the following information, where applicable:

- Accession codes, unique identifiers, or web links for publicly available datasets
- A list of figures that have associated raw data
- A description of any restrictions on data availability

The authors declare that all data supporting the findings of this study are available within this published article and its Supplementary Information files and from the corresponding author upon reasonable request.
The source data underlying Figs 1c, 3a, 5e-f, 6m, 7h-k and Supplementary Figs 5a-b, 7b; and 11b, d and f are provided as Source Data File.
In situ hybridization data used in Figs. 1a, b and Supplementary Figs 1a, b and 3c are available from the Allen Developing Mouse Brain Atlas (www.alleninstitute.org).
In situ hybridization data used in Supplementary Figs 5a-d and 10a-b are available from the Eurexpress atlas (www.eurexpress.org).
The mass spectrometry proteomics data have been deposited to the ProteomeXchange Consortium via the PRIDE partner repository64 with the dataset identifier PXD011918 [http://www.ebi.ac.uk/pride/archive/projects/PXD011918]. Main and supplementary figures have been deposited in Figshare depository [https://doi.org/10.6084/m9.figshare.7588481.v2].

Field-specific reporting

Please select the one below that is the best fit for your research. If you are not sure, read the appropriate sections before making your selection.

☒ Life sciences ☐ Behavioural & social sciences ☐ Ecological, evolutionary & environmental sciences

For a reference copy of the document with all sections, see nature.com/documents/nr-reporting-summary-flat.pdf

Life sciences study design

All studies must disclose on these points even when the disclosure is negative.

Sample size	Sample size was determined according to the availability of biological samples with n=3 as the minimal number of biologically independent samples used in each experiment. This number of biological replicates represents a minimal standard sample size used commonly in the biological sciences.
Data exclusions	No data were excluded from the analysis.
Replication	All the images presented in main or supplementary figures are representative of at least 3 independently repeated and successful attempts at verification.
Randomization	Randomization was not relevant in this study, since no experimental setting presented in the manuscript required random allocation of samples/organisms/participants into experimental groups.
Blinding	The investigators were blinded to group allocation during data analysis.

Reporting for specific materials, systems and methods

We require information from authors about some types of materials, experimental systems and methods used in many studies. Here, indicate whether each material, system or method listed is relevant to your study. If you are not sure if a list item applies to your research, read the appropriate section before selecting a response.

Materials & experimental systems

n/a	Involved in the study	
☐	☒	Antibodies
☐	☒	Eukaryotic cell lines
☒	☐	Palaeontology
☐	☒	Animals and other organisms
☒	☐	Human research participants
☒	☐	Clinical data

Methods

n/a	Involved in the study	
☒	☐	ChIP-seq
☒	☐	Flow cytometry
☒	☐	MRI-based neuroimaging

Antibodies

Antibodies used	Beta-actin (4970, Cell Signalling); APOA1 (B-5257, LSBio); APOB (20737, Abcam); APOE (B6780, LSBio); APOE (SC-6384, Santa Cruz); APOJ (AF2747, R&D); AQP1 (SC-55466, Santa Cruz); Axin1 (2087, Cell Signaling); Active Beta-Catenin (05-565, Millipore); CD63 (SC-15353, Santa Cruz); Claudin-1 (51-9000, Invitrogen); DVL2 (CS-3216, Cell Signalling); DVL3 (SC-8027, Santa Cruz); Flotillin-2 (610383, BDBioscience); GAPDH (CS-5174, Cell Signalling); Golgin97 (A21270, Invitrogen); HA (ab9110, Abcam); HA (SC-7392, Santa Cruz); HSP70 (SC-24, Santa Cruz); ROR1 (kind gift from Henry Ho); TSG101 (HPA006161, Sigma); V5 (R960-25, Invitrogen); Wls (SC-133635, Santa Cruz); Wnt5a (AF645, R&D); Wnt5a (MAB645, R&D); ZO-1 (R26.4C, DSHB).
Validation	Beta-actin (4970, Cell Signalling) - Validated species, applications and references are available at the manufacturer's website. Antibody has been used in the manuscript for WB application in mouse and human samples.<br>APOA1 (B-5257, LSBio) – Validated species, applications and references are available at the manufacturer's website. Antibody has been used in the manuscript for WB and IF application in mouse and human samples. Antibody has been validated for IF using Ig primary antibody as a negative control (Supplementary Fig. 7). For WB antibody specificity has been validated against several other apolipoproteins of human or mouse origin (Supplementary Fig. 8).<br>APOB (20737, Abcam) - Validated species, applications and references are available at the manufacturer's website. Antibody has been used in the manuscript for WB and IF application in mouse and human samples. Antibody has been validated for IF using Ig primary antibody as a negative control (Supplementary Fig. 7). For WB antibody specificity has been validated against several other apolipoproteins of human or mouse origin (Supplementary Fig. 8).<br>APOE (B6780, LSBio) – Antibody has been discontinued by the manufacturer. Antibody has been used in the manuscript in IF application in mouse samples. Antibody has been validated for IF using Ig primary antibody as a negative control (Supplementary Fig. 7).<br>APOE (HPA068768, Sigma) - Validated species, applications and references are available at the manufacturer's website. Antibody

has been used in the manuscript for WB application in human samples. For WB antibody specificity has been validated against several other apolipoproteins of human or mouse origin (Supplementary Fig. 8).
APOE (SC-6384, Santa Cruz) - Antibody has been discontinued by the manufacturer. Antibody has been used in the manuscript for WB application in human mouse samples. Antibody has been validated for IF using Ig primary antibody as a negative control Supplementary Fig. 7).
APOJ (AF2747, R&D) - Validated species, applications and references are available at the manufacturer's website. Antibody has been used in the manuscript for WB, IP and IF application in mouse and human samples. Antibody has been validated for IF using Ig primary antibody as a negative control (Supplementary Fig. 7). For WB antibody specificity has been validated against several other apolipoproteins of human or mouse origin (Supplementary Fig. 8).
AQP1 (SC-55466, Santa Cruz) - Validated species, applications and references are available at the manufacturer's website. Antibody has been used in the manuscript for WB and IF application in mouse and human samples.
Axin1 (CS-2087, Cell Signalling) - Validated species, applications and references are available at the manufacturer's website. Antibody has been used in the manuscript for WB application in mouse samples.
Active Beta-Catenin (05-565, Millipore) - Validated species, applications and references are available at the manufacturer's website. Antibody has been used in the manuscript for WB application in mouse samples.
CD63 (SC-15353, Santa Cruz) - Antibody has been discontinued by the manufacturer. Antibody has been used in the manuscript for WB and IF application in mouse samples.
Claudin-1 (51-9000, Invitrogen) - Validated species, applications and references are available at the manufacturer's website. Antibody has been used in the manuscript for WB application in mouse samples.
DVL2 (CS-3216, Cell Signalling) - Validated species, applications and references are available at the manufacturer's website. Antibody has been used in the manuscript for WB application in mouse samples.
DVL3 (SC-8027, Santa Cruz) - Validated species, applications and references are available at the manufacturer's website. Antibody has been used in the manuscript for WB application in mouse samples.
Flotillin-2 (610383, BDBioscience) - Validated species, applications and references are available at the manufacturer's website. Antibody has been used in the manuscript for WB application in mouse samples.
GAPDH (CS-5174, Cell Signalling) - Validated species, applications and references are available at the manufacturer's website. Antibody has been used in the manuscript for WB application in mouse samples.

Golgin97 (A21270, Invitrogen) - Validated species, applications and references are available at the manufacturer's website. Antibody has been used in the manuscript for WB application in mouse samples.
HA (ab9110, Abcam) - Applications and references are available at the manufacturer's website. Antibody has been used in the manuscript for IP application.
HA (SC-7392, Santa Cruz) - Applications and references are available at the manufacturer's website. Antibody has been used in the manuscript for WB application.
HSP70 (SC-24, Santa Cruz) - Validated species, applications and references are available at the manufacturer's website. Antibody has been used in the manuscript for WB application in mouse samples.
ROR1 (kind gift from Henry Ho) – Validated application and species can be found in Ho et al., 2012. Antibody has been used in the manuscript for WB application in mouse samples.
TSG101 (HPA006161, Sigma) - Validated species, applications and references are available at the manufacturer's website. Antibody has been used in the manuscript for IF and WB application in mouse samples.
V5 (R960-25, Invitrogen) - Applications and references are available at the manufacturer's website. Antibody has been used in the manuscript for WB and IP application.
Wls (SC-133635, Santa Cruz) -
Wnt5a (AF645, R&D) - Validated species, applications and references are available at the manufacturer's website. Antibody has been used in the manuscript for IP application in mouse samples. IP application specificity was validated using IgG as a negative control.
Wnt5a (MAB645, R&D) - Validated species, applications and references are available at the manufacturer's website. Antibody has been used in the manuscript for IF and WB application in human and mouse samples. Antibody has been extensively validated for IF and WB applications using full and conditional KO mouse strains samples.
ZO-1 (R26.4C, DSHB) - Applications and references are available at the manufacturer's website. Antibody has been used in the manuscript for IF and WB applications in mouse samples.

Eukaryotic cell lines

Policy information about cell lines

Cell line source(s)	HEK293T cell line - source: ATCC; MEF (CF-1) cell line - source: ATCC; TR-CSFB - source: kind gift from Juliane Kläs, University of Heidelberg, L-Wnt3a cell line: ATCC , L-Wnt5a cell line: ATCC
Authentication	HEK293T, MEF (CF-1), L-Wnt3a and L-Wnt5a cell lines were purchased from the ATCC database which guarantee their authenticity. TR-CSFB cell line was not authenticated. All cell lines were used with low passage number.
Mycoplasma contamination	All cell lines were tested for presence of mycoplasma and were either negative or displayed very low level of mycoplasma contamination.
Commonly misidentified lines (See ICLAC register)	*Name any commonly misidentified cell lines used in the study and provide a rationale for their use.*

Animals and other organisms

Policy information about studies involving animals; ARRIVE guidelines recommended for reporting animal research

Laboratory animals	WT embryos were obtained from CD1 IGS adult pregnant female mice (Crl:CD1(ICR) mice, Charles River Laboratories, Germany). Wnt5a KO embryos were obtained from Wnt5atm1Amc (www.jax.org) adult pregnant female mice. Wnt5a cKO embryos were obtained from adult pregnant female mice of newly generated Wnt5a conditional knock-out (Wnt5a x FoxJ1-CreERT2 cKO) mouse strain (The Czech Centre for Phenogenomics, Prague, Czech republic).
Wild animals	*Provide details on animals observed in or captured in the field; report species, sex and age where possible. Describe how animals were caught and transported and what happened to captive animals after the study (if killed, explain why and describe method; if released, say where and when) OR state that the study did not involve wild animals.*
Field-collected samples	*For laboratory work with field-collected samples, describe all relevant parameters such as housing, maintenance, temperature, photoperiod and end-of-experiment protocol OR state that the study did not involve samples collected from the field.*
Ethics oversight	Manipulation with CD1 IGS and Wnt5atm1Amc mice was approved by Stockholm's Norra Djurförsörsektiska Nämnd - ethical permit: N158/15, N326/12. Work with Wnt5a x FoxJ1-CreERT2 mouse strain was approved by Central Commission for Animal Welfare of Ministry of Agriculture Czech Republic - ethical permit: PP-90-2015.

Note that full information on the approval of the study protocol must also be provided in the manuscript.
